# Effects of Laser Peripheral Iridotomy in Subgroups of Primary Angle Closure Based on Iris Insertion

**DOI:** 10.1155/2015/581719

**Published:** 2015-08-13

**Authors:** Sung-Cheol Yun, Ji Wook Hong, Kyung Rim Sung, Jin Young Lee

**Affiliations:** ^1^Department of Clinical Epidemiology and Biostatistics, Asan Medical Center, College of Medicine, University of Ulsan, Seoul 138736, Republic of Korea; ^2^Department of Ophthalmology, Asan Medical Center, College of Medicine, University of Ulsan, Seoul 138736, Republic of Korea

## Abstract

*Purpose*. To investigate the effect of laser peripheral iridotomy (LPI) in subgroups of primary angle closure based on iris insertion configuration. *Methods*. Anterior segment optical coherence tomography (AS-OCT) images were obtained before and two weeks after LPI. Qualitative classification of angle closure eyes according to iris insertion (basal insertion group (BG) and nonbasal insertion group (NBG)) was performed. Anterior chamber depth (ACD), lens vault (LV), iris curvature, iris area, iris thickness (IT_750_), and angle opening distance (AOD_750_) 750 microns from scleral spur were calculated. Uni- and multivariate regression analysis was carried out to evaluate factors associated with AOD_750_ before and after LPI. *Results*. Ninety-two eyes of 92 subjects were categorized as NBG (39 eyes) or BG (53 eyes). The mean change after LPI was not significantly different between two groups in all parameters. In both groups, AOD_750_ was affected by ACD (*p* < 0.001, *p* = 0.044) before LPI. AOD_750_ was affected by LV (*p* = 0.012) in NBG, but by ACD (*p* < 0.001) and IT_750_ (*p* = 0.039) in BG after LPI. *Conclusions*. The outcomes of LPI are not significantly different between angle closure subgroups with different iris insertions. However, factors affecting AOD_750_ show differences between two subgroups after LPI.

## 1. Introduction

Primary angle closure glaucoma (PACG) is one of the leading causes of visual loss in Asians [1–3]. Previous studies have reported the several anatomical features of the eyes with PACG including short axial length and shallow anterior chamber [4–7].

In the past, angle closure was entirely diagnosed** by **gonioscopic examination. Gonioscopic examination is still the reference standard for primary angle closure (PAC) diagnosis, but recent advances in imaging devices have allowed various anterior segment (AS) parameters to be measured. AS optical coherence tomography (AS-OCT) quantitatively measures AS parameters using a noncontact method with the subject in a sitting position [8, 9]. Among the several AS parameters, iris-related parameters have become a focus of recent studies. Wang et al. reported that iris curvature (IC), iris area (IA), and iris thickness (IT) are independently associated with the existence of a narrow angle [10, 11]. Furthermore, in several recent papers the peripheral iris thickness was reported as an important predictor of the successful outcome of laser peripheral iridotomy (LPI) [12, 13].

Another point of interest is the location of the iris insertion into ciliary body. The area of the peripheral iris insertion into the ciliary body is very close to the trabecular meshwork, and, thus, characteristics of iris insertion may affect the configuration of the anterior chamber angle and the amount of pupillary block. LPI is performed to open the closed anterior chamber angle by resolving the pupillary block in PAC eyes. Hence, we intended to evaluate whether the effect of LPI is different in subgroups of PAC based on iris insertion configuration using AS-OCT images.

## 2. Methods

### 2.1. Subjects

PAC suspect (PACS) or PAC patients who visited the glaucoma clinic of Asan Medical Center, Seoul, Korea, were seen by a single glaucoma specialist (Kyung Rim Sung) and met the inclusion criteria below which were consecutively included in this study based on a medical record review. The study was approved by the Institutional Review Board of Asan Medical Center and followed the tenets of the Declaration of Helsinki. All participants underwent a complete ophthalmic examination, including a review of their medical history, measurement of best-corrected visual acuity (BCVA), slit-lamp biomicroscopy, Goldmann applanation tonometry, gonioscopy, fundoscopic examination using a 90- or 78-diopter lens, stereoscopic optic disc photography, retinal nerve fiber layer photography, a visual field (VF) test (Humphrey field analyzer, Swedish Interactive Threshold Algorithm (SITA) 24-2; Carl Zeiss Meditec, Dublin, CA), Cirrus HD-OCT (Carl Zeiss Meditec), and AS-OCT (Visante OCT, Carl Zeiss Meditec).

PACS and PAC were diagnosed by gonioscopic examination. Eyes with appositional contact between the peripheral iris and the posterior trabecular meshwork of greater than 270° were included in the PACS group [14]. Eyes with an occludable angle and exhibiting features indicating trabecular obstruction by the peripheral iris were considered to have PAC [14]. PAC was considered present when an eye had an occludable angle (appositional contact between the peripheral iris and the posterior trabecular meshwork of >270°) and exhibited features indicative of trabecular obstruction by the peripheral iris, such as elevated intraocular pressure (IOP), iris whorling (distortion of radially orientated iris fibers), “glaukomflecken” lens opacity, or excessive pigment deposition on the trabecular surface, but without the development of a glaucomatous optic disc or any VF change [14]. We combined both PACS and PAC eyes and defined them as having “angle closure” for our current analysis in particular. Only reliable VF test results (false-positive errors <15%, false-negative errors <15%, and fixation loss <20%) were included in the analysis. Eyes with peripheral anterior synechiae (PAS) in anterior chamber (AC) angle were excluded. We excluded patients with a history or current use of topical or systemic medications (antihistamines, antiepileptics, antiparkinsonian agents, antispasmolytic drugs, mydriatic agents, and sympathetic agents) that could affect the angle or the pupillary reflex [15]; those with a history of previous intraocular surgery (including cataract surgery, laser trabeculoplasty, laser iridoplasty, and laser iridotomy); and those unable to fixate prior to the AS-OCT examination. Among the abovementioned criteria for PAC, patients with a history of acute PAC, defined by the presence of ocular or periocular pain, nausea, or vomiting, and a history of intermittent blurring of vision with haloes; an intraocular pressure (IOP) >30 mmHg; and the presence of at least three of the following: conjunctival injection, corneal epithelial edema, middilated unreactive pupil, and shallow AC, were also excluded [16]. Eyes diagnosed with secondary angle closure, such as those with neovascular or uveitic glaucoma, were also excluded. All eyes were newly diagnosed cases, and AS-OCT imaging was performed before starting any glaucoma medication, laser treatment, or intraocular surgery.

### 2.2. Gonioscopy

Prior to AS-OCT imaging, all patients underwent a slit-lamp examination and gonioscopy, conducted by an independent observer (Kyung Rim Sung) who has extensive experience in the performance of such examinations. All eyes were examined using a Sussman lens in a darkened room (0.5 cd/m^2^). Both static gonioscopy and dynamic gonioscopy were performed using a Sussman lens with the eye in the primary gaze position. Indentation gonioscopy was performed to determine whether angle closure was attributable to apposition or to PAS. Care was taken to ensure that light did not fall on the pupil during the examinations.

### 2.3. AS-OCT Imaging

All participants were imaged in terms of the nasal and temporal angle (0–180°) using AS-OCT (Visante OCT, version 2.0; Carl Zeiss Meditec) operating in the enhanced AS single mode (scan length 16 mm; 256 A-scans). To confirm the consistency of the iris root insertion according to the pupillary reaction, four sessions using four different standardized lighting conditions (3.25, 100.8, 426, and 1420 cd/m^2^), grading from dark to light, was performed by a single well-trained operator. The room in which AS-OCT imaging was performed had four-graded lighting controlled by four-leveled switches. The lighting condition was changed by turning the switch at each session. Thus, the same four light level conditions were provided to all participants. Participants were asked to sit back after imaging and wait for 30 seconds, during which the lighting conditions were changed. After 30 seconds of adaptation to the new lighting conditions, imaging was resumed. Thus, four images, obtained under four different lighting conditions, were obtained from each participant. Among the four images obtained in each session, the images obtained at 3.25 cd/m^2^ were used for the analysis [17]. AS parameters in each image were evaluated by an independent examiner (Ji Wook Hong) who was blind to all other test results and the clinical information for the participants.

**Figure 1:**
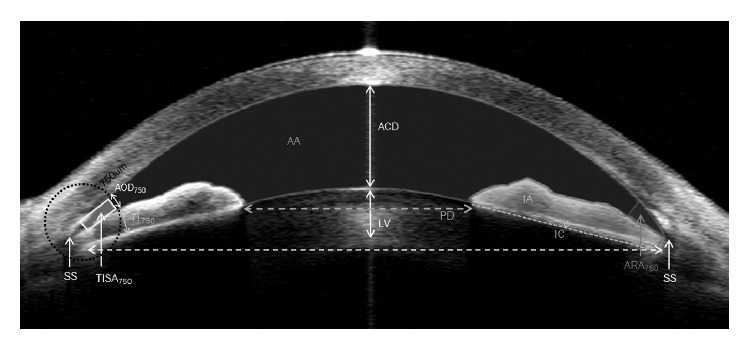
Anterior segment parameters determined by anterior segment optical coherence tomography. Abbreviation: ACD: anterior chamber depth, SS: scleral spur, IT750: iris thickness 750 *μ*m from the scleral spur, IA: cross-sectional area of iris, AA: anterior chamber area, IC: iris curvature, LV: lens vault, AOD_750_: angle opening distance 750 *μ*m anterior to the scleral spur, ARA_750_,: angle recess area, triangular area formed by the AOD_750_, TISA_750_: trabecular-iris space area—trapezoidal area with the following boundaries; anteriorly, the AOD_750_ and posteriorly, a line drawn from the scleral spur perpendicular to the plane of the inner sclera wall to the opposing iris; superiorly, the inner corneoscleral wall; and inferiorly, the iris surface, PD: pupillary distance.

All parameters were determined using Image J software (ver. 1.44, National Institutes of Health, Bethesda, MD). The analyzed parameters are described in Figure 1. Anterior chamber depth (ACD) was defined as the distance from the corneal endothelium to the anterior surface of the lens. The scleral spur was defined as the point at which a change in curvature of the inner surface of the angle wall became apparent and often presented as an inward protrusion of the sclera [18]. After determination of the scleral spur location, iris thickness 750 *µ*m from the scleral spur (IT_750_) was measured [11]. Iris area (IA) was defined as the cross-sectional area of the iris. Anterior chamber area (AA) was defined as the cross-sectional area bordered at the corneal endothelium and anterior surface of the lens and iris. Iris curvature (IC) was defined as the maximum perpendicular distance between the iris pigment epithelium and a line connecting the most peripheral to the most central point of the epithelium [11]. The lens vault (LV) was defined as the perpendicular distance between the anterior pole of the crystalline lens and a horizontal line joining the two scleral spurs [19]. Angle opening distances (AOD_750_) which were defined as the linear distance between the point of the inner corneoscleral wall (750 *µ*m anterior to the scleral spur) and the iris, were also assessed. The ARA_750_ was defined as the triangular area formed by the AOD_750_. The corners of the triangle were the angle recess (the apex), the iris surface, and the inner corneoscleral wall. TISA_750_ was defined as the trapezoidal area with the following boundaries: anteriorly, the AOD_750_; posteriorly, a line drawn from the scleral spur perpendicular to the plane of the inner scleral wall to the opposing iris; superiorly, the inner corneoscleral wall; and, inferiorly, the iris surface. Measurement variability of the parameters was checked prior to the full analysis by calculating the intraclass correlation coefficients (ICCs). Intraexaminer ICC values for various AS parameters ranged between 0.933 and 0.951 [20].

**Figure 2:**
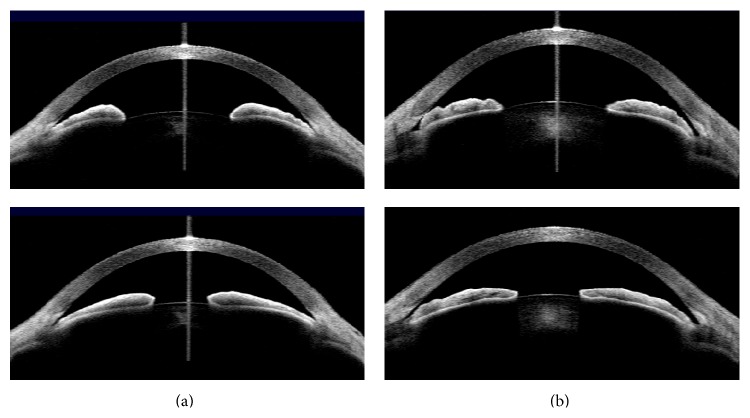
Categorization of primary angle closure eyes according to the location of iris root insertion. (a) Basal insertion (upper; dark, lower; light lighting conditions). (b) Nonbasal insertion (upper; dark, lower; light lighting conditions).

The image acquisition procedure and analysis methods have been previously described in detail [20–22]. All parameters except for ACD, LV, AA, and pupillary distance (PD) were measured on both nasal and temporal sides and the average of the two values was used for analysis. Iris root insertion configuration was independently assessed by two glaucoma experts (Kyung Rim Sung and Jin Young Lee) who were blind to other AS-OCT parameters and all other test results including the clinical information of the participants. Four images at different lighting conditions were reviewed by two experts. Iris root insertion was categorized into two groups, a nonbasal group (NBG) and a basal insertion group (BG), according to the presence of a space between scleral spur and iris root (Figure 2). Each grader classified each eye as NBG or BG. If the opinions of the two observers differed, the eye in question was excluded.

### 2.4. Laser Peripheral Iridotomy (LPI)

LPI was performed in the superior region of the iris (from 10 to 2 o'clock) by sequential argon and neodymium-yttrium-aluminum-garnet laser after pretreatment with 2% pilocarpine instilled into the eye one hour before the LPI. The power settings used were 500–1000 mW with a spot size of 50 *µ*m for a duration of 0.05 seconds with the argon laser and 2–5 mJ with the yttrium-aluminum-garnet laser. Topical medications that could affect the angle measurement were not prescribed at the post-LPI.

### 2.5. Statistical Analysis

The Wilk-Shapiro test was used to explore the distribution of the numerical data. An unpaired Student's *t*-test was used for comparisons between NBG and BG of age, baseline IOP, spherical equivalent (SE), Cirrus HD OCT measured retinal nerve fiber layer (RNFL) thickness, and PD. Categorical variables were compared by Chi square test. We used a mixed-effects regression to calculate the pre-post AS-OCT parameter's mean difference and relative mean difference, the latter defined by (preparameter − postparameter)/preparameter × 100. For each outcome we also calculated pre-post outcome change adjusted for age, gender, SE, PD, and the pre-post PD difference. Residual diagnostic plots were used to detect features of concern in the model. Exploratory analyses of the residuals suggested that the chosen models were appropriate for all parameters. Univariate and multivariate regression analysis was performed to evaluate the factors associated with AC angle narrowing in each group. Univariate analyses were performed separately for each variable. Variables with a probability value ≤0.20 in univariate analyses were included in the multivariate analysis. AC angle narrowing was defined as AOD_750_. All reported *p*-values were two-sided, and a value of *p* < 0.05 was considered statistically significant. SAS software, version 9.2 (SAS Institute, Inc., Cary, NC) and SPSS version 15.0 (SPSS Inc., Chicago, IL), was used for the statistical analyses.

## 3. Results

Ninety-two PAC (60) or PACS subjects (32) were imaged and subcategorized as NBG (39 eyes) or BG (53 eyes). Three eyes were excluded due to different opinions between graders regarding the assessment AS-OCT image. All were East Asians (77 women: 15 men). There was a significant difference in age between the groups, with NBG subjects being older (62.7 ± 5.7 versus 59.8 ± 7.3 years, *p* = 0.043). NBG eyes were more hyperopic (1.29 ± 1.17 versus 0.813 ± 1.00 diopter, *p* = 0.039). The baseline IOP was marginally higher in BG eyes (16.6 ± 5.2 versus 14.8 ± 3.4 mmHg, *p* = 0.063). The average RNFL thickness and VF mean deviation were not different between the two groups. The demographic features and baseline status of the study subjects are listed in Table 1.

The mean differences of the AS-OCT parameters obtained at pre- and post-LPI were not significantly different between the BG and NBG eyes. In addition, there were no differences in the percentage changes in any parameter between the two groups after LPI (Table 2).

In both groups, AOD_750_ was affected by ACD (NBG; *p* < 0.001, BG; *p* = 0.044) before LPI (Tables 3 and 4). However, anatomical factors affecting the AOD_750_ did show a difference between the two groups after LPI. AOD_750_ was affected by LV (*p* = 0.012) in NBG (Table 5) but by ACD (*p* < 0.001) and IT_750_ (*p* = 0.039) in BG after LPI (Table 6).

## 4. Discussion

The mechanism of angle closure involves the interplay between anatomic predisposition and physiological factors. Recent studies of anterior chamber parameters obtained by AS-OCT have led to the identification of several novel anatomic risk factors for angle closure, such as increased iris thickness and area, greater lens vault, and smaller anterior chamber width [10, 11, 19, 23]. Moreover, physical variations of the iris and ciliary body structures may play a role in the development of angle closure. It is conceivable that basal iris insertion contributes to angle crowding more than nonbasal insertion and, thus, predisposes an eye with crowded anterior chamber characteristics (such as a short axial length [24, 25], smaller anterior chamber width [23], or greater lens vault [19]) to pupillary block and subsequent PAC. In our current study, we aimed to categorize PAC eyes according to the configuration of iris insertion into the ciliary body and to analyze whether the effect of the LPI is different in PAC subgroups based on iris insertion. Also, we investigated anatomic risk factors for angle closure in such subgroups based on iris insertion characteristics.

Iris insertion was categorized in our study into two groups, NBG and BG, according to the presence of a space between the scleral spur and the peripheral side of the basal iris. NBG and BG subjects had some different features; that is, NBG cases were older and hyperopic. The IOP was marginally higher in BG eyes. Interestingly, the mean change after an LPI was not significantly different between our two groups in any AS-OCT parameter, nor did the percentage changes differ between the two groups in any parameter. In other words, pupillary block is considered to exist in both groups, and thus the effect of pupillary block on angle closure might not be different between the two groups, since LPI was expected to resolve the pupillary block. Additionally, factors that affect the angle narrowing were rather similar in the two groups prior to the LPI, showing that ACD was the most important factor for angle narrowing. However, factors affecting angle narrowing were different between the two groups after the LPI. In the NBG cases, a greater LV was associated with angle narrowing while a thicker peripheral iris was associated with the BG. The mean age of the NBG was older than that of the BG. Aging is reported to significantly increase LV, and a higher LV may play an important role in the mechanism of angle closure [22]. This effect may result from the induction of the forward movement of the lens due to zonular laxity or increases in lens thickness, which can cause an elevated LV. Also, increased LV can directly induce narrowing of the peripheral angle or increase pupillary block by expanding iridolenticular contact.

A thicker peripheral iris was found to be associated with a narrow angle in the BG eyes after a resolution of the pupillary block, indicating that this would contribute to angle crowding in these cases. A thicker peripheral iris is likely to contribute to angle closure, because the peripheral iris would be in closer proximity to the angle [10]. This finding supports the concept that increased thickness and bulk of the iris root anterior to the plane of the scleral spur push the peripheral iris against the trabecular meshwork, thereby worsening angle crowding in an already predisposed eye.

Multiple pathogenic mechanisms are expected to contribute to PAC. The outcomes of LPI differed between angle closure subgroups with different anatomical characteristics, suggesting that the pathogenic mechanism of angle closure may differ among subgroups [26]. In previous studies, a considerable portion of the PAC eyes analyzed showed a closed angle despite a successful LPI, and those eyes that underwent LPI showed progressive narrowing afterwards [22, 25, 27]. These earlier studies suggested that the nonpupillary block mechanism may substantially contribute to PAC. Hence, predicting which factor is more important in the development of angle closure in specific eyes would be beneficial. Our current results may provide some clue that the contributing factors may be different according to the iris insertion configurations.

The limitations of our study must be acknowledged. First, although two experienced clinicians qualitatively graded iris insertion and peripheral iris configuration, subjective identification of anatomic landmarks could lead to inaccurate determination of reference points and may result in misclassification. However, we tried to minimize the variability by using standard photographs for comparison and to create a consensus between the two graders. In a dilated state, the iris root insertion was not differentiated in some eyes, so we acquired 4 serial images with different lighting conditions and reviewed all 4 images to determine the location of the iris root insertion. By performing image acquisitions at 4 light levels, we believe that we reduced the possibility of misclassification.

Second, AS-OCT images have a limited resolution and some features such as the position of the ciliary processes and iris angulation are thus difficult to identify with this modality. In this context, ultrasound biomicroscopy (UBM) offers better imagery, and several previous publications have nicely categorized PAC using UBM [27–29]. Those studies categorized iris insertion configurations into three categories (apical, mid, and basal configuration). Since AS-OCT does not show the whole feature of the ciliary body, discerning an apical versus mid insertion was difficult. We instead used two classifications: basal and nonbasal insertions. Finally, the relatively small sample size we used may have affected our ability to detect subtle differences in the AS-OCT parameters.

## 5. Conclusions

Our current findings indicate that the outcome of the LPI shows no significant difference between angle closure subgroups classified according to the iris root insertion into the ciliary body. However, anatomical factors affecting the AOD_750_ do show a difference between these two subgroups after LPI. This suggests that identification of the iris insertion may provide some clues to further understanding the anatomical factors that contribute to angle closure after LPI.

## Figures and Tables

**Table 1:** Demographic features and baseline status of the study subjects.

	NBG (*n* = 39)	BG (*n* = 53)	*p*-value
Age (years)	62.7 ± 5.7	59.8 ± 7.3	0.043^†^
Sex (male/female)	5/34	10/43	0.217^‡^
Spherical equivalent (diopter)	1.29 ± 1.17	0.81 ± 1.00	0.039^†^
Baseline IOP (mmHg)	14.8 ± 3.4	16.6 ± 5.2	0.063^†^
VF MD (decibel)	−2.60 ± 2.99	−2.26 ± 4.03	0.662^†^
Average RNFL thickness (micron)	77.1 (±35.9)	82.7 (±29.3)	0.419

Abbreviations: BG; basal insertion group, NBG; nonbasal insertion group, IOP; intraocular pressure, VF MD; visual field mean deviation, RNFL; retinal nerve fiber layer; †: *t*-test, ‡: Chi-square test.

**Table 2:** Mean difference in the AS-OCT parameters in the two study groups at pre- and post-LPI.

	Pre-LPI		Post-LPI		*p*-value (difference)	*p*-value (difference, ratio)
	NBG	BG	NBG	BG		
ACD (mm)	2.100 ± 0.211	2.110 ± 0.301	2.092 ± 0.208	2.113 ± 0.307	0.3624	0.4208
LV (mm)	0.982 ± 0.200	0.889 ± 0.245	1.071 ± 0.234	0.967 ± 0.266	0.1444	0.1511
IC (mm)	0.354 ± 0.077	0.320 ± 0.076	0.131 ± 0.059	0.104 ± 0.045	0.9582	0.2412
IA (mm^2^)	1.516 ± 0.268	1.588 ± 0.235	1.632 ± 0.297	1.674 ± 0.246	0.9107	0.8769
ARA_750_ (mm^2^)	0.154 ± 0.081	0.116 ± 0.079	0.193 ± 0.070	0.149 ± 0.088	0.8848	0.1536
TISA_750_ (mm^2^)	0.122 ± 0.063	0.099 ± 0.067	0.164 ± 0.058	0.132 ± 0.073	0.7420	0.0568
AOD_750_ (mm)	0.229 ± 0.083	0.239 ± 0.098	0.314 ± 0.102	0.284 ± 0.136	0.0584	0.2082
IT_750_ (mm)	0.359 ± 0.065	0.378 ± 0.082	0.369 ± 0.067	0.381 ± 0.066	0.5110	0.6178

^∗^A mixed-effects model was used to compare the pre-post AS-OCT parameter's mean difference and relative difference between the two groups, adjusted for age, gender, SE, PD and the difference of pre-post-PD.

Abbreviations: AS-OCT: anterior segment optical coherence tomography; BG: basal insertion group; NBG: nonbasal insertion group; ACD: anterior chamber depth; IT_750_: iris thickness from the scleral spur (at 750 m from the scleral spur); IA: cross-sectional area of the iris; IC: iris curvature; LV: lens vault; AOD_750_: angle opening distances (corneoscleral wall 750 m anterior to the scleral spur); ARA_750_: angle recess area formed by the AOD_750_; TISA_750_: trabecular-iris space area, trapezoidal area with the following boundaries: anteriorly, the AOD_750_; posteriorly, a line drawn from the scleral spur perpendicular to the plane of the inner sclera wall to the opposing iris; superiorly, the inner corneoscleral wall; and inferiorly, the iris surface; PD: pupillary distance.

**Table 3:** Uni- and multivariate linear regression analysis of the association between various parameters and anterior chamber angle narrowing (AOD_750_) assessed pre-LPI in the NBG subjects.

	Univariate			Multivariate		
	SE	*B* coefficient (95% CI)	*p*-value	SE	*B* coefficient (95% CI)	*p*-value
ACD, mm	0.052	0.218 (0.113, 0.323)	<0.001	0.052	0.218 (0.113, 0.323)	<0.001
LV, mm	0.057	−0.084 (−0.216, 0.048)	0.207			
IC, mm	0.171	−0.132 (−0.478, 0.215)	0.293			
IA, mm^2^	0.048	−0.050 (−0.147, 0.047)	0.300			
IT_750_, mm	0.200	0.219 (−0.187, 0.626)	0.281			
PD, mm	0.020	−0.010 (−0.051, 0.031)	0.614			

Abbreviations: NBG: nonbasal insertion group; ACD: anterior chamber depth; IT_750_: iris thickness from the scleral spur (at 750 m from the scleral spur); IA: cross-sectional area of the iris; IC: iris curvature; LV: lens vault; AOD_750_: angle opening distances (corneoscleral wall 750 m anterior to the scleral spur); PD: pupillary distance; SE: standard error; CI: confidence interval; LPI: laser peripheral iridotomy.

**Table 4:** Uni- and multivariate linear regression analysis of the association between various parameters and anterior chamber angle narrowing (AOD_750_) assessed pre-LPI in the BG subjects.

	Univariate			Multivariate		
	SE	*B* coefficient (95% CI)	*p*-value	SE	*B* coefficient (95% CI)	*p*-value
ACD, mm	0.048	0.092 (−0.005, 0.190)	0.063	0.087	0.174 (0.007, 0.333)	0.044
LV, mm	0.061	−0.075 (−0.203, 0.051)	0.235			
IC, mm	0.177	−0.141 (−0.497, 0.215)	0.430			
IA, mm^2^	0.053	−0.091 (−0.198, 0.017)	0.096	0.045	−0.084 (−0.175, 0.007)	0.071
IT_750_, mm	0.162	0.050 (−0.277, 0.376)	0.761			
PD, mm	0.018	−0.020 (−0.056, 0.016)	0.268			

Abbreviations: BG: basal insertion group; ACD: anterior chamber depth; IT_750_: iris thickness from the scleral spur (at 750 m from the scleral spur); IA: cross-sectional area of the iris; IC: iris curvature; LV: lens vault; AOD_750_: angle opening distances (corneoscleral wall 750 m anterior to the scleral spur); PD: pupillary distance; SE: standard error; CI: confidence interval; LPI: laser peripheral iridotomy.

**Table 5:** Uni- and multivariate linear regression analysis of the association between various parameters and anterior chamber angle narrowing (AOD_750_) assessed post-LPI in the NBG subjects.

	Univariate			Multivariate		
	SE	*B* coefficient (95.0% CI)	*p*-value	SE	*B* coefficient (95.0% CI)	*p*-value
ACD, mm	0.077	0.168 (0.011, 0.325)	0.036	0.086	0.076 (−0.098, 0.251)	0.381
LV, mm	−0.191	−0.191 (−0.322, −0.059)	0.006	0.065	−0.191 (−0.305, −0.040)	0.012
IC, mm	0.286	0.111 (−0.469, 0.691)	0.701			
IA, mm^2^	0.053	−0.098 (−0.206, 0.010)	0.075	0.051	−0.071 (−0.174, 0.031)	0.168
IT_750_, mm	0.250	−0.284 (−0.792, 0.223)	0.263			
PD, mm	0.019	−0.012 (−0.027, 0.051)	0.540			

Abbreviations: NBG: nonbasal insertion group; ACD: anterior chamber depth; IT_750_: iris thickness from the scleral spur (at 750 m from the scleral spur); IA: cross-sectional area of the iris; IC: iris curvature; LV: lens vault; AOD_750_: angle opening distances (corneoscleral wall 750 m anterior to the scleral spur); PD: pupillary distance; SE: standard error; CI: confidence interval; LPI: laser peripheral iridotomy.

**Table 6:** Uni- and multivariate linear regression analysis of the association between various parameters and anterior chamber angle narrowing (AOD_750_) assessed post-LPI in the BG subjects.

	Univariate			Multivariate		
	SE	*B* coefficient (95.0% CI)	*p*-value	SE	*B* coefficient (95.0% CI)	*p*-value
ACD, mm	0.092	0.185 (0.000, 0.370)	0.050	0.050	0.217 (0.116, 0.318)	<0.001
LV, mm	0.104	−0.059 (−0.270, −0.151)	0.572			
IC, mm	0.459	−0.261 (−1.184, 0.662)	0.572			
IA, mm^2^	0.099	−0.051 (−0.249, 0.148)	0.608			
IT_750_, mm	0.280	−0.483 (−1.047, 0.080)	0.091	0.235	−0.500 (−0.973, −0.028)	0.039
PD, mm	0.028	−0.003 (−0.059, 0.053)	0.918			

Abbreviations: BG: basal insertion group; ACD: anterior chamber depth; IT_750_: iris thickness from the scleral spur (at 750 m from the scleral spur); IA: cross-sectional area of the iris; IC: iris curvature; LV: lens vault; AOD_750_: angle opening distances (corneoscleral wall 750 m anterior to the scleral spur); PD: pupillary distance; SE: standard error; CI: confidence interval; LPI: laser peripheral iridotomy.
